# Development and Evaluation of Docetaxel-Phospholipid Complex Loaded Self-Microemulsifying Drug Delivery System: Optimization and In Vitro/Ex Vivo Studies

**DOI:** 10.3390/pharmaceutics12060544

**Published:** 2020-06-12

**Authors:** Miao Wang, Sung-Kyun You, Hong-Ki Lee, Min-Gu Han, Hyeon-Min Lee, Thi Mai Anh Pham, Young-Guk Na, Cheong-Weon Cho

**Affiliations:** 1 College of Pharmacy and Institute of Drug Research and Development, Chungnam National University, 99, Daehak-ro, Yuseong-gu, Daejeon 34134, Korea; wmclare@163.com (M.W.); skyou@hanall.co.kr (S.-K.Y.); hk_lee@cnu.ac.kr (H.-K.L.); linuxfalcon@naver.com (M.-G.H.); gusals2218@naver.com (H.-M.L.); phammaianhdkh68@gmail.com (T.M.A.P.); 2 Hanall Biopharma Co. Ltd., 199 Techno 2-ro, Yuseong-gu, Daejeon 34025, Korea

**Keywords:** docetaxel, phospholipid complex, self-microemulsifying drug delivery system, dissolution, cell uptake, permeability

## Abstract

Docetaxel (DTX) has clinical efficacy in the treatment of breast cancer, but it is difficult to develop a product for oral administration, due to low solubility and permeability. This study focused on preparing a self-microemulsifying drug delivery system (SME) loaded with DTX-phospholipid complex (DTX@PLC), to improve the dissolution and gastrointestinal (GI) permeability of DTX. A dual technique combining the phospholipid complexation and SME formulation described as improving upon the disadvantages of DTX has been proposed. We hypothesized that the complexation of DTX with phospholipids can improve the lipophilicity of DTX, thereby increasing the affinity of the drug to the cell lipid membrane, and simultaneously improving permeability through the GI barrier. Meanwhile, DTX@PLC-loaded SME (DTX@PLC-SME) increases the dissolution and surface area of DTX by forming a microemulsion in the intestinal fluid, providing sufficient opportunity for the drug to contact the GI membrane. First, we prepared DTX@PLC-SME by combining dual technologies, which are advantages for oral absorption. Next, we optimized DTX@PLC-SME with nanosized droplets (117.1 nm), low precipitation (8.9%), and high solubility (33.0 mg/g), which formed a homogeneous microemulsion in the aqueous phase. Dissolution and cellular uptake studies demonstrated that DTX@PLC-SME showed 5.6-fold higher dissolution and 2.3-fold higher DTX uptake in Caco-2 cells than raw material. In addition, an ex vivo gut sac study confirmed that DTX@PLC-SME improved GI permeability of DTX by 2.6-fold compared to raw material. These results suggested that DTX@PLC-SME can significantly overcome the disadvantages of anticancer agents, such as low solubility and permeability.

## 1. Introduction

Docetaxel (DTX), an anticancer agent, is one of the second generation taxoids. It leads to apoptosis of malignant cells by inducing cell death [1]. DTX is widely known as an effective anticancer agent for breast, ovarian, lung, and hormonal refractory prostate cancers, but is limited in clinical use, due to several side effects such as hypersensitivity responses, bone marrow suppression, alopecia, stomatitis, and mucositis [2,3]. The commercial product (Taxotere^®^) is an intravenous formulation, which is generally used for clinical use, but the parenteral route can cause infection, phlebitis, infiltration, extravasation, fluid overload, hypothermia, electrolyte imbalance, etc. [4,5]. The formulation may lower patient compliance, due to the inconvenience of administration. Oral formulations can be a suitable alternative to intravenous formulations, as they reduce multiple side effects including pain, allergic reactions, and infections and promote patient compliance.

However, the development of oral formulation has been difficult, because of the low oral bioavailability (<5%) of DTX [6]. The BCS class IV drug, DTX, is partially insoluble in water, and has low permeability across the gastrointestinal (GI) epithelium, due to P-glycoprotein (P-gp) mediated efflux, which limits the GI absorption and oral bioavailability of DTX [7]. Therefore, the oral delivery of DTX requires pharmaceutical techniques to overcome these adverse properties of DTX.

Phospholipid complexes that form molecular complexes between phospholipids and drugs can be proposed as an alternative technique for developing oral formulations of DTX [8,9]. This technique, DTX-phospholipid complex (DTX@PLC), increases the permeation through the membrane of the intestinal epithelium much more easily, thereby facilitating the GI absorption of DTX [10]. It has been reported that the phospholipid complex can significantly enhance both the bioavailability and efficacy of DTX [10]. However, the phospholipid complex exhibits disadvantages, such as poor solubility and dispersibility in the aqueous phase, mainly due to increased lipophilicity [11,12]. Therefore, to further increase the bioavailability of DTX via oral administration, it is necessary to introduce additional pharmaceutical techniques that enhance the dissolution performance of DTX@PLC.

Self-microemulsifying drug delivery systems (SME) are often used to increase the dissolution of orally administered drugs [13]. SME formulation is an isotropic mixture of oil, surfactant, and cosurfactant that can be dispersed in GI fluids to form micro- or nano-emulsions with nanosized droplets [14]. The droplet size (100–300 nm) is much smaller than the particle size of raw materials (10–1000 µm), which can significantly improve dissolution and permeability [13]. Moreover, compared to other techniques, SME formulations offer multiple advantages, including ease of manufacture, thermodynamic stability, and low cost. 

Therefore, the SME technique can be applied to improve the low dissolution of phospholipid complexes. A new formulation for DTX@PLC-loaded SME (DTX@PLC-SME), which combines the advantages of phospholipid complex and SME technique, was developed in this study. We hypothesized that the dual technique can be used to improve the dissolution and permeability of DTX. The specific aims of this study are (i) to screen the excipients for selecting the reliable combinations of DTX@PLC-SME, and to optimize the composition of DTX@PLC-SME through design of experiment; (ii) to investigate the characteristics of formulation such as dissolution, cytotoxicity, and cellular uptake; and (iii) to evaluate the permeability of formulation with non-everted rat gut sac model.

## 2. Materials and Methods

### 2.1. Chemicals and Reagents

DTX was provided by Korea United Pharma Inc. (Seoul, Korea). Lauroglycol 90, Lauroglycol FCC, Labrafac Lipophile WL 1349, Labrafac CC, Capryol 90, Labrafil M 1944 CS, Transcutol P, and Labrasol were obtained from Gattefossé Co. (Lyon, France). Capmul MCM was purchased from the Abitec Corporation (Cleveland, OH, USA). Polyethylene glycol 400 (PEG 400), Tween 20, and Tween 80 were bought from Samchun Chemical Co., Ltd (Pyungtaek, Korea). Cremophor EL and Pluronic L64 were provided by BASF Co. (Ludwigshafen, Germany). Tetraglycol, propylene glycol, dimethyl sulfoxide (DMSO), Coumarin-6 (C6), and 3-(4,5-dimethylthiazol-2-yl)-2,5-diphenyltetrazolium bromide (MTT) were purchased from Sigma-Aldrich (St. Louis, MO, USA). Lipoid^®^ S100 (phospholipid, PLC) was obtained from Lipoid Co. (Ludwigshafen, Germany). The high-performance liquid chromatography (HPLC) grade methanol, acetonitrile, *n*-hexane, and ethanol were bought from J.T. Baker (Phillipsburg, NJ, USA).

### 2.2. HPLC Analysis

High-performance liquid chromatography (HPLC) analysis for DTX was conducted with previously reported method [15]. Agilent 1100 series (Agilent Technologies Inc., Santa Clara, CA, USA), equipped with an Xterra RP18 column (250 × 4.6 mm, 5 μm; Waters, Milford, MA, USA) was used for analysis. The temperature of the column was maintained at 45 °C, and the mobile phase was a mixture consisting of acetonitrile and distilled water (45:55, *v*/*v*). The UV-wavelength, flow rate, and injection volume were 230 nm, 1.2 mL/min, and 20 µL, respectively.

### 2.3. Preparation of DTX@PLC

DTX@PLC was fabricated by the solvent evaporation method [8]. The mass ratio of DTX and PLC was determined based on the preliminary screening results (supplementary data). DTX (100 mg) and phospholipid (300 mg) were placed in a round bottom flask, which were completely dissolved in ethanol (20 mL) as a solvent. The mixture was placed in a thermostat shaker (BS-21; Jeio Tech Co., Ltd., Daejeon, Korea) and stirred for 1 h (40 °C and 100 rpm). Then, the solvent was evaporated using a rotary evaporator (Rotavapor R-3; Büchi Labortechnik AG, Flawil, Switzerland). Residues were collected and stored in the freezer until further evaluation.

### 2.4. Preparation of DTX@PLC-SME and DTX-SME

DTX@PLC-SME was prepared by a classic shaking method with slight modification [16]. First, the blank-SME (without drug) was prepared with a combination of selected oil (18.8 *w*/*w*% Capryol 90), surfactant (71.2 *w*/*w*% Cremophor EL), and cosurfactant (10.0 *w*/*w*% tetraglycol), through screening of excipients (Section 2.5) and optimization (Section 2.6) of DTX@PLC-SME. DTX@PLC (10 mg corresponding to 2.5 mg DTX) was placed in a tube containing blank-SME (300 mg), and then shaken for 72 h to reach equilibrium. Simultaneously, DTX-SME was prepared by dissolving DTX (2.5 mg) in the blank-SME (300 mg).

### 2.5. Screening of Excipients for DTX@PLC-SME

#### 2.5.1. Solubility Test

The solubility of DTX@PLC in different excipients was measured according to an equilibrium method [17]. Briefly, an excess amount of DTX@PLC was added to tubes containing 1 g of oils (Labrafac CC, Lauroglycol 90, Lauroglycol FCC, Labrafil M 1944 CS, Labrafac Lipophile WL 1349, Capmul MCM, and Capryol 90), surfactants (Labrasol, Tween 20, Tween 80, Pluronic L64, and Cremophor EL), or cosurfactants (propylene glycol, polyethylene glycol 400, Transcutol P, and tetraglycol). The tubes were sealed and rotated for 72 h to reach equilibrium with an angle rotator (Model AG; Fine PCR, Gunpo, Korea) at 25 °C. The mixtures were then centrifuged at 15,000× *g* for 10 min, to remove undissolved DTX@PLC. The supernatant was diluted with 50 *v*/*v*% propanol, and the concentration of DTX was measured by HPLC analysis.

#### 2.5.2. Emulsification Study

An emulsification study was conducted to select the most suitable surfactant and cosurfactant. In order to form finely dispersed microemulsions, a type IV composition was used [13]. First, the blank-SME formulations consisting of 20% oil, 40% surfactant, and 40% cosurfactant were prepared with the excipients preselected in Section 2.5.1. Subsequently, 100 mg of blank-SMEs were diluted with different pH values of medium and distilled water (10 mL). The mixture was shaken for 10 min to obtain a homogenous microemulsion. The degree of self-emulsification was evaluated in terms of three-standards, including droplet size, transmittance, and phase separation, based on the previously reported grading system (Table 1) [18]. Droplet size (diameter) was measured using an electrophoretic laser scattering (ELS) analyzer (ELS-Z2; Otsuka Electronics, Osaka, Japan) at 25 °C after diluting the formulation (100 mg) with distilled water (10 mL). Phase separation was determined by visual inspection [19].

#### 2.5.3. Pseudo-Ternary Phase Diagram

The composition range of the SME formulation capable of forming microemulsions was established through a pseudo-ternary phase diagram. Based on emulsification studies, the pseudo-ternary phase diagram was constructed by combining Capryol 90 (oil), Cremophor EL (surfactant), and tetraglycol (cosurfactant). The vertices in the diagram represented each excipient. For any mixture, the sum of each excipient was 100%. Mixtures containing different percentages of excipients were prepared and evaluated by the method described in Section 2.5.2. A range of suitable SME formulations belonging to grades A and B was defined and used in further studies.

### 2.6. Experimental Optimization of DTX@PLC-SME

Design Expert^®^ 11 (Stat-Ease Inc., Minneapolis, MN, USA) was used as a tool for designing experiments and statistically analyzing the results. In this study, D-optimal mixture design was applied to optimize the composition of SME formulation [20]. Three components of the SME formulation were set as independent variables. Based on the pseudo-ternary phase diagram, the percentages of Capryol 90 (X_1_), Cremophor EL (X_2_), and tetraglycol (X_3_) were set within the ranges of 10–30%, 10–80%, and 10–80%, respectively (Table 2). The total percentage in all experiments was up to 100%. The solubility of DTX@PLC in SME formulation (Y_1_), precipitation of DTX (Y_2_), droplet size (Y_3_), and transmittance (Y_4_) were set as responses, to optimize the SME formulation. The 17 designed experiments were conducted, and an appropriate fitting model for each response was obtained by comparing various statistical parameters, such as sequential *p*-values, lack of fit, squared correlation coefficient (R^2^), adjusted R^2^, predicted R^2^, and adequate precision [21]. After fitting the statistical model, the desirability values were obtained through numerical optimization considering the goal of response. The composition of DTX@PLC-SME with the highest desirability value was determined as the optimal formulation.

#### 2.6.1. Solubility of DTX@PLC-SME in SME (Y_1_)

The solubility of DTX@PLC in the SME was measured to prepare DTX@PLC-SME capable of dissolving the maximum amount of DTX@PLC with the smallest amount of SME. Briefly, excess DTX@PLC was mixed in the prepared SME (1 g) and rotated for 72 h to reach equilibrium. All samples were centrifuged at 15,000× *g* for 10 min. The supernatant was diluted with 50 *v*/*v*% propanol, and then analyzed with HPLC method.

#### 2.6.2. Precipitation (Y_2_)

Precipitation was investigated to prepare a formulation in which DTX@PLC-SME could form a uniform droplet without precipitation in the aqueous phase. Briefly, distilled water (10 mL) was added to DTX@PLC-SME (100 mg), and then the mixture was vortexed for 30 min to constitute a uniform microemulsion. The microemulsion was filtered using a 0.22 µm filter. The filtrate was diluted with 50 *v*/*v*% methanol and analyzed with HPLC. The precipitation (%) was calculated by the following equation:

$$Precipitation\;\left(\%\right)=100\left(\frac{C_{MeOH}-C_{DW}}{C_{MeOH}}\right)$$

where *C_MeOH_* is the concentration of DTX in DTX@PLC-SME dispersed in methanol. *C_DW_* is the concentration of DTX in DTX@PLC-SME dispersed in distilled water. A precipitation value close to 0% means no precipitation occurs.

#### 2.6.3. Droplet Size (Y_3_)

Droplet size was measured using an ELS analyzer (ELS-Z2; Otsuka Electronics, Osaka, Japan). Briefly, 100 mg of DTX@PLC-SME was added to 10 mL of distilled water and the mixture was gently stirred to obtain a homogenous microemulsion. After placing the mixture in a cuvette, the sample was monitored according to the user manual of ELS analyzer.

#### 2.6.4. Transmittance (Y_4_)

The absorbance was measured with a microplate reader (Sunrise; Tecan Group Ltd.; Männedorf, Switzerland) to obtain transmittance of DTX@PLC-SME. Briefly, DTX@PLC-SME (100 mg) was diluted with distilled water (10 mL) to form a microemulsion. The samples were dispensed into 96-well plates and the absorbance was measured at 620 nm. Distilled water was used as a control. The transmittance was calculated by the following equation:

$$Transmittance\;\left(\%\right)=100\;\times \;10^{\left(Abs_{DW}-Abs_{sample}\right)}$$

where *Abs_DW_* and *Abs_sample_* represent the absorbances of the distilled water and sample, respectively. The value close to 100% means a transparent microemulsion.

#### 2.6.5. Transmission Electron Microscope

The morphology of optimized DTX@PLC-SME was observed with a transmission electron microscope (TEM) (JEM 2100F; JEOL Ltd., Tokyo, Japan). After DTX@PLC-SME was dispersed in distilled water, the sample (10 µL) was placed on a copper grid and dried at room temperature. The sample was confirmed at an acceleration voltage of 120 kV.

### 2.7. Dissolution Test

Dissolution test was conducted according to the United States Pharmacopeia (USP) apparatus II method with a dissolution tester (DST-810; Labfine, Anyang, Korea) [22]. Distilled water and different pH media prepared according to USP guidelines (pH 1.2, pH 4.0, and pH 6.8) were used in the experiment. DTX (raw material), DTX@PLC, DTX-SME, and DTX@PLC-SME equivalent to 10 mg DTX were packed in hard gelatin capsules (00 size). DTX@PLC-SME was prepared by mixing 10 mg of DTX@PLC (2.5 mg DTX) with 300 mg of blank-SME, and DTX-SME was also prepared by adding 2.5 mg DTX to blank-SME in the same procedure. The volume of medium was 900 mL at 37.0 ± 0.5 °C and the speed of paddle was set to 50 rpm. Samples (5 mL) were collected at scheduled time-points (5, 10, 15, 30, 45, 60, 90, and 120 min), and the equal volume of fresh medium was filled. The samples were filtered through a 0.45 µm filter, and then diluted with 50 *v*/*v*% methanol. The concentration of DTX was measured with HPLC analysis.

### 2.8. Cell Studies

#### 2.8.1. Cell Culture

Caco-2 cell line (heterogeneous human epithelial colorectal adenocarcinoma) was purchased from Korean Cell Line Bank (Seoul, Korea). The cells were cultured in Dulbecco’s Modified Eagle’s Medium with 10% fetal bovine serum and 1% penicillin/streptomycin. The condition of incubator was maintained at 37 °C with 5% CO_2_.

#### 2.8.2. Cytotoxicity Study

The cytotoxicity of formulations against Caco-2 cells was assessed by MTT assay. Briefly, cells (5 × 10^4^ cells/well, 100 µL) in a culture medium were distributed in 96-well plates and incubated for 24 h. The cells were treated with DTX-formulations (DTX (raw material), DTX@PLC, DTX-SME, and DTX@PLC-SME corresponding to 0.01–100 μg/mL DTX) and blank-formulations (blank@PLC, blank-SME, and blank@PLC-SME corresponding to 1–500 µg/mL formulation), and then incubated for 24 h. MTT solution (30 µL/well, 5 mg/mL) was added to plates and incubated for an additional 4 h. The medium was removed and DMSO (200 µL) was added to each well, and the absorbance was measured at 565 nm, using a microplate reader (Sunrise; Tecan Group Ltd., Männedorf, Switzerland). Cell viability was calculated according to the following equation:

$$Cell\;viability\;\left(\%\right)=\frac{Abs_{sample}}{Abs_{control}}\times 100$$

where *Abs_sample_* and *Abs_control_* are the absorbance of sample treated and untreated cells, respectively.

#### 2.8.3. Cellular Uptake Study

Cellular uptake of DTX (raw material), DTX@PLC, DTX-SME, and DTX@PLC-SME into Caco-2 cells was evaluated. The cells (1 × 10^6^ cells/well, 3 mL) were dispensed into 6-well plates and incubated for 24 h. The cells were treated with DTX (raw material), DTX@PLC, DTX-SME, and DTX@PLC-SME corresponding to 20 µg/mL DTX. After incubation for 4 h, the wells were washed twice using cold phosphate-buffered saline (PBS). The cells were lysed with 1 *v*/*v*% Triton X-100 solution (0.5 mL/well). Then, acetonitrile (0.5 mL/well) was added to extract DTX from lysed cells and shaken for 5 min. The mixtures were transferred to tubes and centrifuged at 15,000× *g* for 10 min. The supernatant was filtered through a 0.45 μm filter and the concentration of DTX was analyzed by HPLC. The amount of DTX absorbed from different formulations was normalized with the amount of protein measured by bicinchoninic acid assay.

Fluorescence observation was performed to visualize the cellular uptake of DTX@PLC-SME. The hydrophobic dye, C6, was added to the preparation of DTX@PLC, DTX-SME, and DTX@PLC-SME for fluorescence staining. Caco-2 cells (1 × 10^3^ cells/well, 1 mL) were dispensed into 12-well plates and incubated at 37 °C for 24 h. C6 solution as a control and formulations (corresponding to 100 ng/mL C6) were diluted with cell medium containing 1 *v*/*v*% DMSO and treated to each well. After a 4 h incubation, the wells were washed twice with cold PBS, and fixed with 4% formaldehyde for 5 min. Subsequently, cell nuclei were stained with 4′,6-diamidino-2-phenylidone (DAPI) for 5 min. The wells were washed again with cold PBS twice, and the cells were observed using EVOS^TM^ M5000 imaging system (Thermo Fisher Scientific, Waltham, MA, USA). The formulations containing C6 were named as C6-DTX@PLC, and C6-DTX-SME, C6-DTX@PLC-SME.

### 2.9. Ex Vivo Intestinal Permeability Study

#### 2.9.1. Animals

Male Sprague-Dawley rats (Nara-Biotec, Seoul, Korea) weighing 200–250 g were housed in a light control room at 22 °C and a relative humidity of 55%. All animal experiments were conducted by following the protocol (No. CNU-01167) and “Animal Use Guidelines” approved by Chungnam National University Institutional Animal Care and Use Committee (Daejeon, Korea).

#### 2.9.2. Non-Everted Gut Sac Model

Non-everted gut sac models are usually performed to investigate drug permeability through the GI membrane. The experiment was carried out by modifying the previously reported method [23,24]. Briefly, rats (*n* = 4 for each group) were sacrificed by injecting CO_2_ gas and then incised in the middle of the abdomen. Then, the intestinal segment (jejunum) was excised, and immediately washed with a cold Krebs-Ringer solution using a syringe to remove intestinal debris. One end of the segment (5 cm) was carefully tied with a silk suture. After knotting, syringe and tube were connected to the empty segment, and then the segment was filled with DTX (raw material), DTX@PLC, DTX-SME, or DTX@PLC-SME (corresponding to 60 µg/mL DTX dispersed in Krebs-Ringer solution, 1 mL). Subsequently, the segment was placed in a tube containing 50 mL Krebs-Ringer solution at 37 °C and gently stirred at 50 rpm. Gas mixture of 95% O_2_ and 5% CO_2_ was continuously provided to the media during the incubation. Sample (1 mL) was collected from the tube at predetermined time-points (30, 60, 90, 120, and 240 min), and then filled with an equal volume of preheated Krebs-Ringer solution. The area of each segment was measured after the experiment. The collected samples were filtered through a 0.22 µm filter and then analyzed by LC-MS/MS. The flux rate (Δ*Q*/Δ*t*) was obtained from the slope of linear regression of the cumulative DTX amounts permeated through the GI membrane over time. The cumulative amount of DTX permeated (*Q*) and the apparent permeability coefficient (*P_app_*) were calculated by the following equation:

$$Q\left(\mu g\right)=C_{t}V+0.02\overset{n}{\underset{i=1}{\sum }}C_{i}$$

where *Q* is the cumulative DTX amount, *C_t_* is the DTX concentration in the tube at time *t*, *C_i_* is the DTX concentration at previous time-point, *V* is the volume of medium in the tube.

$$P_{app}\left(cm/s\right)=\frac{\Delta Q/\Delta t}{Area\times C_{0}}$$

where *P_app_* is the apparent permeability coefficient, Δ*Q*/Δ*t* is the flux rate, *Area* is the surface area of the segment, and *C*_0_ is the initial concentration of DTX in the segment.

#### 2.9.3. LC-MS/MS Analysis

The concentrations of DTX were analyzed with an LC-MS/MS system consisting of Agilent 1290 series and Agilent 6495 Triple Quad LC/MS (Agilent Technologies, Santa Clara, CA, USA). A YMC-Triart C18 column (50 × 2.0 mm, 1.9 µm; YMC Inc., Wilmington, NC, USA) was used for the analysis. The mobile phase was a mixture of 0.2% formic acid in acetonitrile and 0.2% formic acid in distilled water (50:50, *v*/*v*), which was flowed at a rate of 0.5 mL/min. The temperature of the column and autosampler was maintained at 30 °C and 4 °C, respectively. Scan mass spectra were recorded with the positive ion mode of Agilent jet stream electrospray ionization (AJS-ESI). The ion transitions of DTX and paclitaxel as an internal standard (ISTD) were set as 830.5 → 303.9 *m*/*z* (collision energy: 24 V) and 876.4 → 308.1 *m*/*z* (collision energy: 30 V), respectively, and detected with a multiple reaction monitoring (MRM) mode. Other analysis conditions were set as follows: gas temp 200 °C, gas flow 14 L/min, nebulizer 20 psig, sheath gas flow 11 L/min, sheath gas temp 250 °C, nozzle voltage 1500 V, ion injector 3000 V.

In this study, the lowest quantitative limit (LLOQ) for DTX was 0.5 ng/mL. The calibration curve for DTX was set in the range of 0.5–1000 ng/mL. The curve was plotted with linear regression and showed linearity of *R*^2^ > 0.998. LC-MS/MS data processing was performed with Agilent analysis software (Agilent MassHunter Quantitative Software Version B.07.00; Agilent Technologies, Santa Clara, CA, USA).

### 2.10. Statistical Analysis

All data were represented as mean ± standard deviation (SD). Statistical significance of the differences (*p* < 0.05) was assessed by student’s t-test or one-way analysis of variance (ANOVA) of GraphPad Prism 8.4 (GraphPad Software Inc., La Jolla, CA, USA).

## 3. Results and Discussion

### 3.1. Screening of Excipients for DTX@PLC

**Figure 1:**
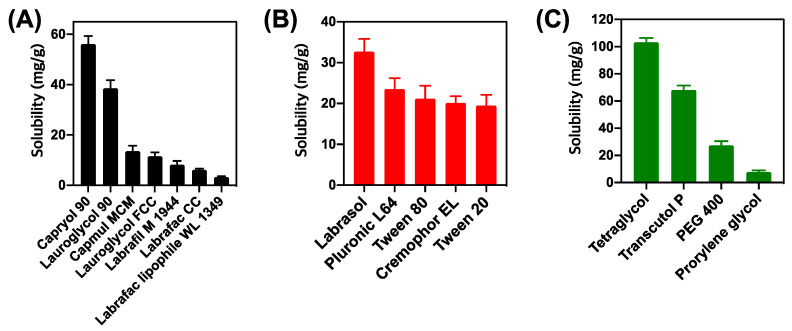
Solubility study of docetaxel-phospholipid complex (DTX@PLC) in various excipients. (**A**) Oils, (**B**) surfactants, and (**C**) cosurfactants. Values are expressed as mean ± SD (*n* = 3).

To prepare the SME formulation that dissolves unit dose of DTX@PLC using a minimal amount of formulation, the first step was to choose the appropriate excipient that can provide a high solubility of DTX@PLC. Oil can dissolve the lipophilic drug and further improve the lipophilicity of the drug [25]. As shown in Figure 1A, Capryol 90 exhibited the highest solubility of DTX@PLC (56.1 mg/g) among the oils. Thus, Capryol 90 was chosen as an oil of SME formulation. For surfactants, all surfactants exhibited suitable solubility for DTX@PLC (>20 mg/g) (Figure 1B). Generally, surfactants lower the surface energy of the interface and disperse the oil into small droplets in the aqueous phase [26]. We tested all surfactants for future emulsification studies. Among the cosurfactants, Transcutol P and tetragylcol represented higher solubility than other surfactants (103.2 and 68.2 mg/g, respectively) (Figure 1C). Cosurfactant increases the capacity of micelles, preventing drug precipitation and maintaining stable micelles [27]. Hence, Transcutol P and tetragycol were selected as surfactants for further studies.

The self-emulsifying ability of the preselected excipients was investigated. SME formulations were prepared by combining surfactants and cosurfactants. As listed in Table 3, the combination of Capryol 90, Cremophor EL, and tetraglycol showed excellent self-emulsifying ability (grade A or B) than other combinations such as Capryol 90, Tween 20, and tetraglycol. Additionally, no phase separation was observed in this combination. The emulsifying efficiency of surfactant can vary, depending on the hydrophilic-lipophilic balance, chemical structure, and chain length, as well as the structure of the oil tested. For cosurfactants, tetraglycol is more hydrophilic than Transcutol P, and a cosurfactant with high hydrophilicity may have the ability to emulsify an oil-surfactant mixture in contact with aqueous phase more quickly and effectively. Based on these results, Capryol 90, Cremophor EL, and tetraglycol were chosen as suitable excipients for the preparation of DTX@PLC-SME.

**Figure 2:**
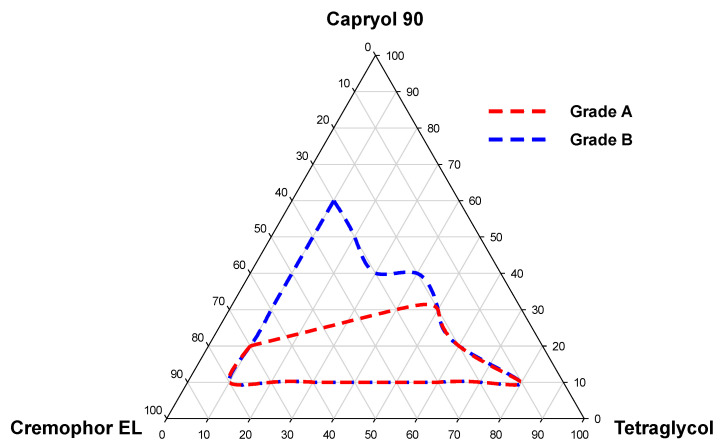
Pseudo-ternary phase diagram consisting of Capryol 90, Cremophor EL, and tetraglycol. The red and blue lines indicate grade A and B microemulsions, respectively.

Next, we plotted a pseudo-ternary phase diagram to set the content range of excipients. In Figure 2, the red and blue lines represent microemulsions with transparent appearance (grade A) and a slightly less clear appearance (grade B), respectively. An unstable emulsion was formed in a range, in which the oil percentage exceeds 30%. In addition, the SME formulations containing surfactants and cosurfactants in the range of 10–80% could form emulsions with droplets of less than 300 nm. Therefore, in the further study, optimization of DTX@PLC-SME was conducted in the range of 10–30% oil and 10–80% surfactant/cosurfactant.

### 3.2. Optimization of DTX@PLC-SME

D-optimal mixture design was used for optimizing DTX@PLC-SME [20]. Statistical analysis suggested that the relationship between factors and responses based on different fitting models. The percentage of Capryol 90 (X_1_), Cremophor EL (X_2_), and tetraglycol (X_3_) were set as factors. The solubility of DTX@PLC in SME (Y_1_), precipitation (Y_2_), droplet size (Y_3_), and transmittance (Y_4_) were set as responses to evaluate DTX@PLC-SME, which could achieve a stable and improved formulation. DTX@PLC-SME with high Y_1_ value can load large amounts of DTX@PLC with minimal SME amount, improving productivity and dosing convenience [13]. A low Y_2_ value indicates that the formulation forms a stable microemulsion in the aqueous phase [28]. DTX@PLC-SME with a low Y_3_ value can increase the contact with the GI membrane, due to the large surface area and improve the oral absorption of DTX by permeating the membrane through the intracellular and/or intercellular pathways [17]. In addition, a high Y_4_ indicates that DTX@PLC-SME can form a homogenous and transparent microemulsion [15].

The statistical models and parameters derived based on the interaction of each factor for predicting responses are listed in Table 4. Statistical models for Y_1_, Y_2_, Y_3_, and Y_4_ were proposed as linear, special quadratic, linear, and cubic models, respectively. Statistical parameters such as *p*-value, lack of fit, *R*^2^, adjusted *R*^2^, and predicted *R*^2^ were investigated as indicators for determining the fit of statistical models [14]. The sequential *p*-value indicated that the suggested model was significant at a 95% confidence level, which was less than 0.05 in all models. The lack of fit *p*-values were more than 0.05, which indicated that the models were adequate for explaining the relationship between factor and response [29]. The *R*^2^ and adjusted *R*^2^ values reflected the match between the model-based responses and the experimental data. The *R*^2^ and adjusted *R*^2^ values of responses except Y_3_ were more than 0.9, indicating that the observed data were similar to the model-based responses [30]. Although *R*^2^ value of Y_3_ was 0.7361, the adequate precision value of Y_3_ was 10.2590, which suggests that the Y_3_ response could be applied to the design space (adequate precision > 4) [13]. Additionally, the differences between *R*^2^ and adjusted *R*^2^ in all responses were less than 0.2.

**Figure 3:**
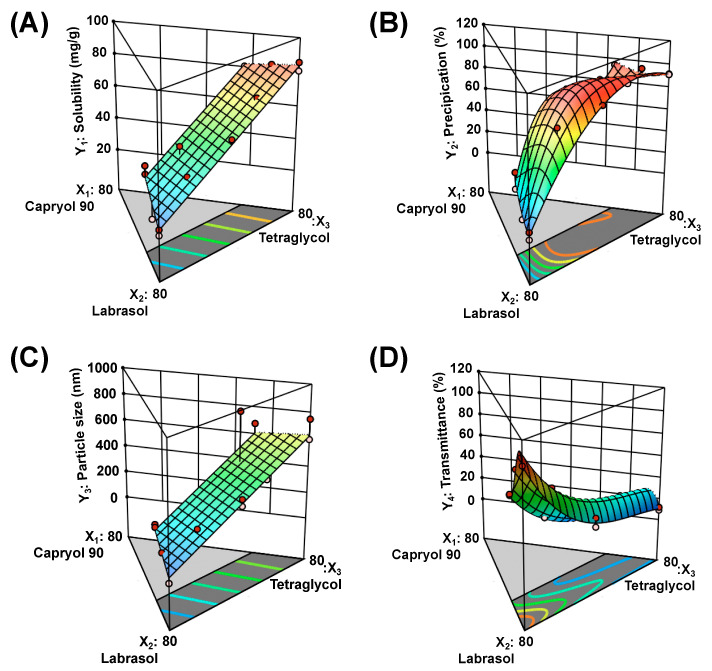
Three-dimensional response surface plots of (**A**) Y_1_: solubility of DTX@PLC in a self-microemulsifying drug delivery system (SME), (**B**) Y_2_: precipitation, (**C**) Y_3_: droplet size, and (**D**) Y_4_: transmittance. The red and blue colors indicate high and low Y values, respectively.

The relationships between factors were explained with three-dimensional plots (Figure 3) and coded equations (Table S1). Table 5 represents the observations of designed compositions. The ranges of Y_1_, Y_2_, Y_3_, and Y_4_ values were from 20.8 to 85.7 mg/g, 0.1 to 94.8%, 11.9 to 855.4 nm, and 4.9 to 99.9%, respectively. In Table S1, the positive and negative coefficients mean synergistic and antagonistic effects on the responses, respectively. In the case of Y_1_, the highest coefficient of X_3_ indicated that X_3_ had the most influence on Y_1_ among the factors. In Y_2_ and Y_3_, the interaction of X_2_ and X_3_ had the greatest impact. As X_3_ increased, the values of Y_2_ and Y_3_ increased. In the case of Y_4_, the value decreased as X_2_ increased, and the interaction effect by all factors was confirmed.

**Figure 4:**
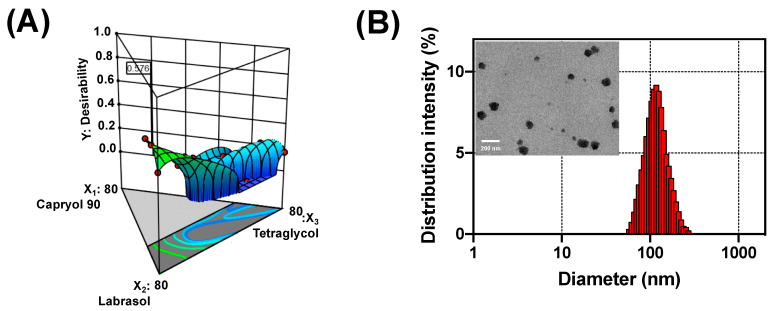
(**A**) Desirability plot through numerical optimization. (**B**) Droplet size and transmission electron microscope (TEM) image of optimal docetaxel-phospholipid complex loaded self-microemulsifying drug delivery system (DTX@PLC-SME). Scale bar is 200 nm.

The composition of DTX@PLC-SME was optimized through a desirability function. Figure 4 shows the desirability plot obtained from numerical optimization. The X_1_, X_2_, and X_3_ values of optimal formulation were 18.8%, 72.2%, and 10.0%, respectively, and the desirability value was 0.576. Table 6 lists the predicted and observed responses of optimal DTX@PLC-SME.

The solubility (Y_1_) and precipitation (Y_2_) of DTX@PLC-SME were 33.0 mg/g and 8.9%, respectively. Capryol 90 (X_1_) and tetraglycol (X_3_) improved the stability of the formulation by providing a high solubility capacity [31]. And, the optimal DTX@PLC-SME formed a homogeneous and transparent microemulsion. The droplet size (Y_3_) and transmittance (Y_4_) of the optimal formulation were 117.1 nm and 96.0%, respectively. In Figure 4B, the particle size measured from ELS analyzer is similar with that observed from TEM image. The nanosized and homogenous droplets may be associated with the amphiphilic surfactant, Cremophor EL, in the formulation, and the hydrophobic and hydrophilic groups were distributed in combination with the hydrophobic group of DTX@PLC and the aqueous phase, respectively [32]. This phenomenon would reduce the interfacial tension and provide sufficient coverage to the surface of the droplets [33].

In order to assess the accuracy of prediction, the differences between the predicted and observed values for all responses were calculated with the following equation: error percentage = [observed value − predicted value] × 100/predicted value [34]. The error percentage of less than 10% means that the accuracy of the optimization is appropriate, and the percentages for all responses fall within 10% (0.9–8.2%). In addition, all observations of the optimal formulation were included in the 95% confidence interval of the predictions. In summary, we optimized the composition of DTX@PLC-SME through statistical design. Subsequent studies were conducted with the optimal formulation.

### 3.3. Dissolution Test

**Figure 5:**
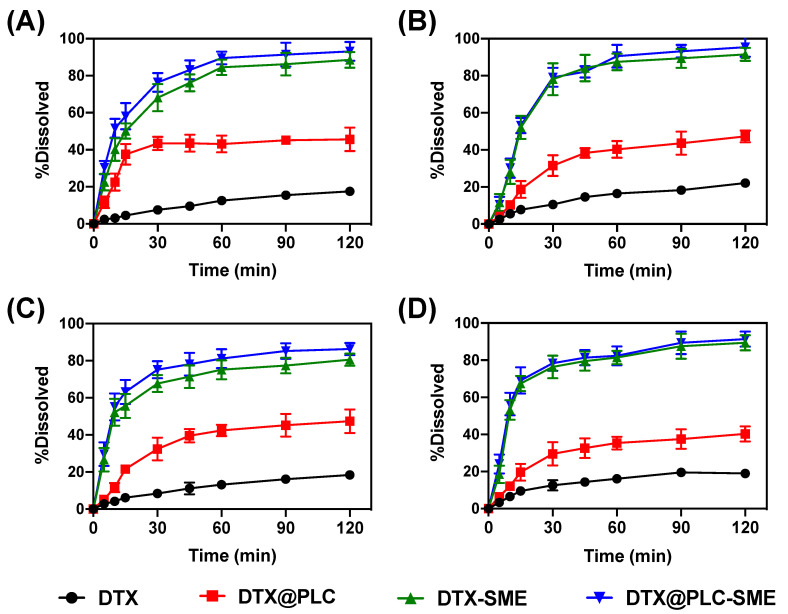
Dissolution profiles of DTX, DTX@PLC, docetaxel-loaded self-microemulsifying drug delivery system (DTX-SME), and DTX@PLC-SME in various media. (**A**) pH 1.2; (**B**) pH 4.0; (**C**) pH 6.8; (**D**) distilled water. Values are presented as mean ± SD (*n* = 3).

In the present experiment, we assumed that the dissolution of DTX might be remarkably improved by phospholipid complexation and/or SME formulation. As shown in Figure 5, DTX shows a low dissolution (<25%) in different media. In the case of DTX@PLC, the dissolution was slightly higher than the raw material (DTX), resulting in a dissolution of 35–50% at 2 h. The result might be due to the solubilizing effect by the phospholipid complex. Comparing to DTX, DTX-SME revealed a dramatic enhancement of the dissolution in different media. DTX@PLC-SME with dual technology also substantially improved the dissolution over 80% within 1 h. These results may be obtained for the following reasons: (i) the emulsifying ability of SME formulation leads to increased distribution, wettability, and drug solubilization in the diffusion layer, thereby improving the dissolution of DTX [35]; (ii) in accordance with the amorphous form of DTX@PLC described in Figure S2 (supplementary data), it may have higher thermodynamic activity than crystalline form, prompting high dissolution of DTX; (iii) the nanosized droplets and thus large surface areas cause drug supersaturation to maintain high dissolution during subsequent periods [36]. Consequently, the substantial enhancement of dissolution was caused by SME technology, as well as the phospholipid complex.

### 3.4. Cell Studies

#### 3.4.1. Cytotoxicity Study

**Figure 6:**
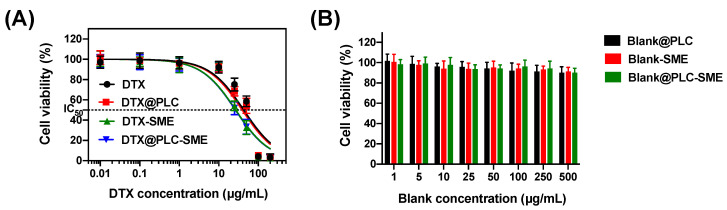
(**A**) Cytotoxicity of docetaxel (DTX), DTX@PLC, docetaxel-loaded self-microemulsifying drug delivery system (DTX-SME), and DTX@PLC-SME on Caco-2 cells for 24 h. Values are presented as mean ± SD (*n* = 6). The dotted marks and solid lines represent the observed data and the nonlinear regression curves, respectively. (**B**) Cytotoxicity of blank-formulations (without drug) on Caco-2 cells for 24 h. Values are presented as mean ± SD (*n* = 6).

Cytotoxicity against Caco-2 cells was assessed to investigate the toxicity of formulations in the GI tract. Caco-2 cells differentiate into monolayers with tight junctions and transport systems, and are generally used as in vitro models that mimic the intestinal environment. Cell viability above 70% is generally considered ‘non-toxic’ and less than 50% is considered ‘irritant’ [37]. Figure 6A represents the cell viability of formulations containing DTX. DTX concentration-dependent cytotoxicity was confirmed in all formulations, and DTX@PLC-SME showed similar toxicity to DTX (raw material). IC_50_ values of DTX and DTX@PLC-SME were 35.8 and 26.4 µg/mL, respectively (*p* > 0.05) (Table S7 in Supplementary Data). For blank-formulations, cytotoxicity showing less than 50% viability was not found in the experimental concentration range (Figure 6B). These results indicate that the cytotoxicity was related to DTX concentrations greater than 25 µg/mL rather than blank-formulations. Consequently, DTX@PLC-SME can induce cytotoxicity similar to commercial products with same amount of drug, and the formulation itself is biocompatible and used as a carrier for oral administration.

#### 3.4.2. Cellular Uptake Study

**Figure 7:**
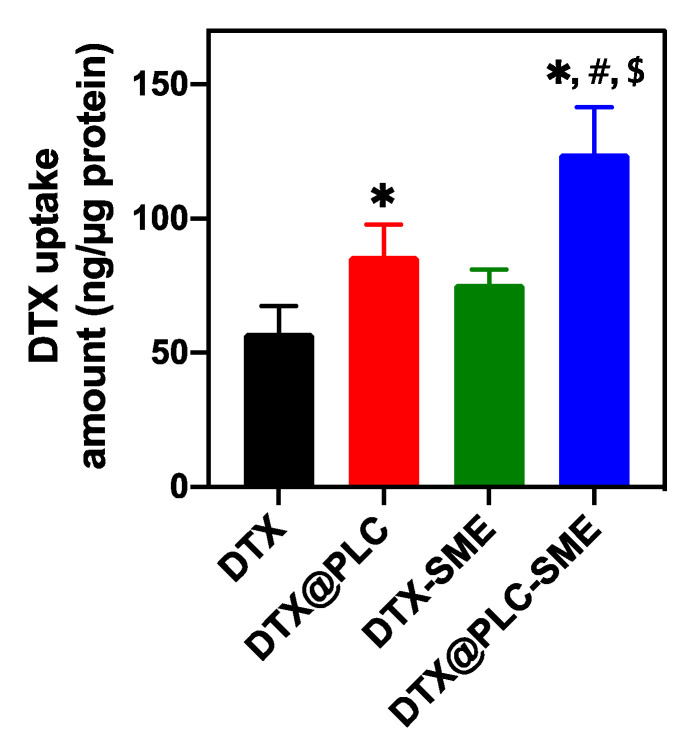
Cellular uptake of DTX, DTX@PLC, DTX-SME, and DTX@PLC-SME into Caco-2 cells. The values are presented as mean ± SD (*n* = 4). * *p* < 0.05 versus DTX. ^#^ *p* < 0.05 versus DTX@PLC. ^$^ *p* < 0.05 versus DTX-SME.

A cellular uptake study was performed, to investigate the effect of DTX@PLC-SME on enhancing DTX uptake into Caco-2 cells. As shown in Figure 7, the cellular uptake of DTX@PLC-SME (124.3 ng/µg) was significantly increased by 2.2-fold, compared to that of raw material (57.4 ng/µg), which was higher than DTX@PLC (86.1 ng/µg) and DTX-SME (75.7 ng/µg) (*p* < 0.05). These results indicated that cellular uptake of DTX was enhanced by phospholipid complexation, as well as SME formulation.

**Figure 8:**
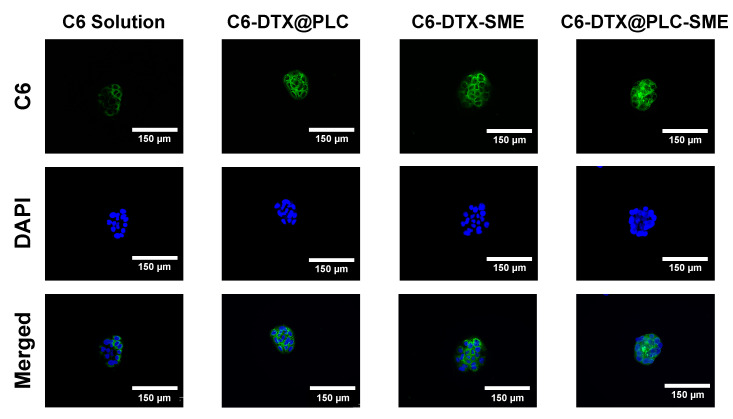
Cellular uptake study in Caco-2 cells observed with fluorescence microscope. The cells were incubated with coumarin-6 (C6) solution (as a control) and C6-loaded formulations, corresponding to 100 ng/mL C6. C6 solution as a control and formulations (corresponding to 100 ng/mL C6) Blue and green colors indicate 4′,6-diamidino-2-phenylidone (DAPI) staining and C6, respectively. The scale bar is 150 µm.

Fluorescence observation was used to visually assess the cellular uptake of the drug in Caco-2 cells (Figure 8). Interestingly, the fluorescence intensity of C6-DTX@PLC and C6-DTX-SME was stronger than that of the C6 solution, indicating that SME formulation, as well as phospholipid complexation can enhance the cellular uptake or accumulation of DTX. C6-DTX@PLC-SME allows more drug to be absorbed into the cell than other formulations, due to its lipophilic, nanoscale, and ability to provide occlusive effects [21]. The large surface area by nanosized droplets can promote the contact between the drug and the cell membrane, thereby enhancing the cellular uptake of drug. In addition, the synergistic effects of phospholipids and lipophilic excipients can improve drug absorption by improving affinity for the cell membranes [18]. Hence, these results supported that the dual technique can overcome the low GI permeation of DTX.

### 3.5. Ex Vivo Intestinal Permeability Study

**Figure 9:**
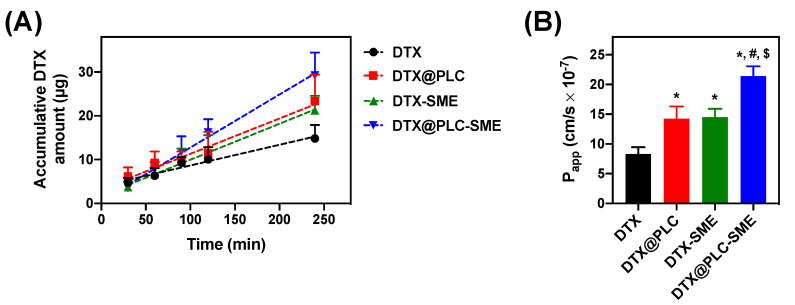
(**A**) Accumulative DTX amount of DTX, DTX@PLC, DTX-SME, and DTX@PLC-SME across the intestinal segment (jejunum) for 240 min. The dotted marks and dashed lines indicate the observed accumulative amounts and the slopes from linear regression analysis with observed values, respectively. Values are presented as mean ± SD (*n* = 4). (**B**) Apparent permeability coefficients (*P*_app_) of DTX, DTX@PLC, DTX-SME, and DTX@PLC-SME investigated with non-everted gut sac model. Values are expresses as the mean ± SD (*n* = 4). * *p* < 0.05 versus DTX. ^#^ *p* < 0.05 versus DTX@PLC. ^$^ *p* < 0.05 versus DTX-SME.

The permeability of DTX, DTX@PLC, DTX-SME, and DTX@PLC-SME was evaluated with a non-everted gut sac model. This model provides clear insights to predict intestinal transport, because intestinal enzyme, transporter expression, and predominance are similar to those of the human intestine [38]. The accumulative DTX permeation was higher in the group of DTX@PLC-SME than in the other groups (Figure 9A). The calculated flux rates of DTX, DTX@PLC, DTX-SME, and DTX@PLC-SME were 47.1, 80.6, 82.1, and 121.0 ng/min, respectively. In addition, the measured P_app_ values of samples were plotted in Figure 9B. The P_app_ of DTX@PLC-SME was 2.6 times higher than that of DTX (*p* < 0.05). In DTX@PLC-SME, the improvement of permeability may be associated with not only the nanoscale and lipophilic droplets but also the inhibition of P-gp efflux.

Cremophor EL constituting DTX@PLC-SME has been reported to inhibit P-gp pumps by altering membrane fluidity or inhibiting substrate binding and efflux pump ATPase [39]. Hence, DTX@PLC-SME has additional advantages in overcoming drug resistance, and in improving the intracellular penetration of DTX belonging to the P-gp substrate. Therefore, the results were derived from the synergistic effect to the nanosized droplets and the inhibitory effect on P-gp efflux, which leads to an improvement in the permeability of DTX in the GI tract.

In summary, DTX@PLC-SME with dual technology consisting of the phospholipid complex and the SME technique could improve the dissolution, absorption, and permeability of DTX. The improvements can be explained for several reasons. First, the increased lipophilicity by phospholipid complexation improves paracellular and intracellular transports, thereby increasing the intestinal absorption and protecting the drug against degradation by intestinal enzymes [40]. Second, nanosized droplets by SME formulation increase dissolution, providing immediate release of the drug, while avoiding migration of drug to unabsorbable distal parts of GI tract [41]. Third, the concentration gradient increases the direct contact between the droplets and the GI membrane, providing a high permeability of the drug [42]. In contrast to raw material, DTX@PLC-SME, due to the synergistic effect of phospholipid complex and SME formulation, enhances the dissolution and permeability of DTX, and in this way may also increase the oral bioavailability.

## 4. Conclusions

DTX@PLC-SME was developed in this study to solve the limitations of DTX, due to its poor solubility and low permeability. DTX@PLC was successfully fabricated by a solvent evaporation method. We also found that SME technology could also greatly improve the dissolution and GI absorption of DTX through simple preparation. By combining the phospholipid complex with SME formulation, DTX@PLC-SME was developed and optimized through the design of experiments. Compared to raw material, DTX@PLC-SME could improve the dissolution of DTX in various media, due to the synergistic effect of dual technology. Cellular uptake and non-everted gut sac studies also demonstrated an improved permeability of DTX, suggesting that the dual technology may enhance the preclinical and clinical effects of DTX. In conclusion, we explored the therapeutic potential of DTX@PLC-SME for overcoming the limitations of DTX at the in vitro/ex vivo level. However, pharmacokinetic and pharmacodynamic studies still need to be further investigated in the next studies.

## Figures and Tables

**Table 1:** Classification standards of emulsification grade.

Grade	Emulsification Capacity	Droplet Size (nm)	Transmittance (%)	Appearance
A	Excellent	<100	>95	Clear and transparent
B	Good	100–300	85–95	Slightly less clear
C	Fair	300–1000	50–85	Milky or grayish white
D	Poor	>1000	<50	No homogeneity

**Table 2:** Factors and responses of D-optimal mixture design.

Factors	Low Limit (*w*/*w*%)	High Limit (*w*/*w*%)
X_1_: Capryol 90	10	30
X_2_: Cremophor EL	10	80
X_3_: Tetraglycol	10	80
**Responses**		**Goal**
Y_1_: Solubility (mg/g)		Maximize
Y_2_: Precipitation (%)		Minimize
Y_3_: Droplet size (nm)		Minimize
Y_4_: Transmittance (%)		Maximize

**Table 3:** The results of emulsification study.

2:4:4 (*w*/*w*%)			Media							
Oil	Surfactant	Cosurfactant	Water		pH 1.2		pH 4.0		pH 6.8	
			Grade	Phase Separation	Grade	Phase Separation	Grade	Phase Separation	Grade	Phase Separation
Capryol 90	Tween 20	Transcutol P	A/B	X ^a^	B	X	B	X	B	X
		Tetraglycol	A/B	X	B	X	B	X	B	X
	Tween 80	Transcutol P	B	X	B/C	X	B	X	B/C	X
		Tetraglycol	A/B	X	B	X	B	X	B	X
	Labrasol	Transcutol P	C/D	X	C/D	O	C/D	O	C/D	O
		Tetraglycol	C/D	X	C/D	O	C/D	O	C/D	O
	Pluronic L64	Transcutol P	C/D	O ^b^	D	O	D	O	D	O
		Tetraglycol	D	O	D	O	C/D	O	D	O
	Cremophor EL	Transcutol P	A/B	X	B	X	B	X	B	X
		Tetraglycol	A/B	X	A	X	A	X	A/B	X

^a^ No phase separation occurs. ^b^ Phase separation occurs.

**Table 4:** Statistical models and parameters of D-optimal mixture design.

Responses	Suggested Model	Model *p*-Value	Lack of Fit *p*-Value	*R* ^2^	Adjusted *R*^2^	Adequate Precision
Y_1_: Solubility	Linear	<0.0001	0.0789	0.9694	0.9650	34.5639
Y_2_: Precipitation	Special quadratic	0.0057	0.2571	0.9872	0.9744	22.0896
Y_3_: Droplet size	Linear	<0.0001	0.2788	0.7361	0.6984	10.2590
Y_4_: Transmittance	Cubic	0.0005	0.7260	0.9973	0.9938	47.3457

**Table 5:** The composition of DTX@PLC-SME and observed responses through D-optimal mixture design. Values are presented as mean ± SD (*n* = 3).

Run	Factors			Responses			
	X_1_	X_2_	X_3_	Y_1_	Y_2_	Y_3_	Y_4_
	Capryol 90	Cremophor EL	Tetraglycol	Solubility	Precipitation	Droplet size	Transmittance
	(*w*/*w*%)	(*w*/*w*%)	(*w*/*w*%)	(mg/g)	(%)	(nm)	(%)
1	30	60	10	45.7 ± 0.8	35.2 ± 19.9	219.4 ± 19.7	55.5 ± 0.2
2	25	50	25	52.7 ± 0.6	84.7 ± 3.2	241.5 ± 28.5	29.7 ± 0.6
3	30	10	60	80.4 ± 1.1	94.8 ± 1.5	371.0 ± 83.1	4.9 ± 0.2
4	15	25	60	70.7 ± 1.5	91.1 ± 0.4	378.4 ± 53.7	32.7 ± 0.4
5	20	10	70	83.1 ± 1.1	93.6 ± 0.2	529.6 ± 192.2	18.9 ± 0.8
6	15	60	25	42.2 ± 1.0	77.5 ± 6.2	243.9 ± 7.1	66.1 ± 0.5
7	10	45	45	56.8 ± 0.5	87.1 ± 1.1	319.7 ± 23.3	21.5 ± 0.3
8	10	80	10	24.2 ± 0.5	6.7 ± 0.7	11.9 ± 1.1	99.9 ± 0.1
9	30	60	10	40.8 ± 0.5	20.5 ± 11.2	239.6 ± 7.5	56.5 ± 0.4
10	10	45	45	56.5 ± 0.4	87.1 ± 0.8	370.4 ± 25.9	29.6 ± 0.1
11	25	25	50	67.9 ± 1.9	92.3 ± 0.5	855.4 ± 137.3	10.5 ± 0.3
12	20	70	10	22.1 ± 0.2	5.5 ± 2.3	131.0 ± 1.3	87.5 ± 0.1
13	30	10	60	80.6 ± 2.9	94.5 ± 0.1	678.6 ± 54.5	7.7 ± 0.2
14	10	10	80	80.7 ± 0.8	90.6 ± 0.6	748.8 ± 39.2	9.1 ± 6.8
15	30	35	35	54.7 ± 1.3	89.4 ± 0.3	324.2 ± 2.5	20.1 ± 0.1
16	10	10	80	85.7 ± 1.6	91.2 ± 0.2	599.5 ± 35.0	11.6 ± 0.5
17	10	80	10	20.8 ± 0.7	0.1 ± 3.4	12.4 ± 1.0	99.3 ± 0.1

**Table 6:** Predicted and observed values of optimal DTX@PLC-SME. Values are presented as mean ± SD (*n* = 3).

Optimal Factors	Responses	95% CI ^a^ Low Predicted Value	Predicted Value	95% CI High Predicted Value	Observed Value	Error Percentage (%)
X_1_: 18.8%	Y_1_: Solubility (mg/g)	27.1	30.5	34.0	33.0 ± 0.2	8.2
X_2_: 71.2%	Y_2_: Precipitation (%)	–1.7	8.7	19.0	8.9 ± 2.6	2.3
X_3_: 10.0%	Y_3_: Droplet size (nm)	4.8	116.1	227.4	117.1 ± 6.7	0.9
	Y_4_: Transmittance (%)	87.2	92.7	98.3	96.0 ± 0.3	3.6

^a^ Confidence interval (CI).

## Supplementary Materials

The following are available online at https://www.mdpi.com/1999-4923/12/6/544/s1. Table S1: Coefficient equations of responses according to the level of factors, Table S2: Analysis of variance for model of solubility (Y1), Table S3: Analysis of variance for model of precipitation (Y2), Table S4: Analysis of variance for model of droplet size (Y3), Table S5: Analysis of variance for model of transmittance (Y4), Table S6: Correlation coefficient (R2) values of docetaxel (DTX)-formulations in various dissolution models, Table S7: IC_50_ values of docetaxel (DTX), docetaxel-phospholipid complex (DTX@PLC), docetaxel-loaded self-microemulsifying drug delivery system (DTX-SME), and docetaxel-phospholipid complex loaded self-microemulsifying drug delivery system (DTX@PLC-SME), determined by MTT assay, Table S8: Long-term stability of DTX@PLC-SME (*n* = 3), Table S9: Freeze-thaw cycle stability of DTX@PLC-SME (*n* = 3), Table S10: Heating-cooling cycle stability of DTX@PLC-SME (*n* = 3), Figure S1: Solubility and solubility enhancing capacity (SEC) versus mass ratio (docetaxel (DTX):phospholipid), Figure S2: (A) X-ray diffraction (XRD) patterns and (B) Fourier transform infrared spectroscopy (FT-IR) spectra of DTX, phospholipid, physical mixture, and docetaxel-phospholipid complex (DTX@PLC), Figure S3: Residual plots. (A) Y1: solubility; (B) Y2: precipitation; (C) Y3: droplet size; (D) Y4: transmittance.
