# Supplementary material for: Development and Evaluation of Docetaxel-Phospholipid Complex Loaded Self-Microemulsifying Drug Delivery System: Optimization and In Vitro/Ex Vivo Studies
Source: Pharmaceutics. 2020 Jun 12;12(6):544. doi: 10.3390/pharmaceutics12060544 (PMC7357111; doi:10.3390/pharmaceutics12060544)
Supplement: Supplementary file 1 [file pharmaceutics-12-00544-s001.pdf]

pharmaceutics

Supplementary Materials: Development and Evaluation of Docetaxel-Phospholipid Complex Loaded Self-Microemulsifying Drug Delivery System: Optimization and In Vitro/Ex Vivo Studies

1. Supplementary Materials and Methods

1.1. Chemicals and Reagents

Docetaxel (DTX) was supplied by Korea United Pharma Inc. (Seoul, Korea). Lipoid® S100 (phospholipid) was obtained from Lipoid Co. (Ludwigshafen, Germany). The high-performance liquid chromatography (HPLC) grade methanol, acetonitrile, n-hexane, and ethanol were bought from J.T. Baker (Phillipsburg, NJ, USA). Other chemical reagents used in this study were analytical grade.

1.2. Preparation of DTX-Phospholipid Complex

DTX-phospholipid complex (DTX@PLC) was fabricated by solvent evaporation method [1]. DTX and phospholipid were placed in a round bottom flask, and completely dissolved in ethanol as a solvent (20 mL). The mixture was placed in a thermostat shaker (BS-21; Jeio Tech Co., Ltd., Daejoen, Korea) at 40 °C, and stirred at 100 rpm for 1 h. Then, the solvent was evaporated using a rotary evaporator (Rotavapor R-3; Büchi Labortechnik AG, Flawil, Switzerland). The residue was collected and stored in the freezer until further evaluations [2].

1.3. Screening of Mass Ratio for DTX@PLC

The ratio of DTX and phospholipid was screened to establish the optimal ratio of DTX@PLC [3]. Briefly, DTX@PLCs with different ratios (DTX:phospholipid = 1:1–5 mass ratio) were prepared with the described method. *n*-hexane containing 3 *v*/*v*% ethanol was added into the excess amount of DTX@PLC, and the mixture was rotated to extract DTX from the complex at room temperature for 24 h. After that, the mixture was centrifuged at 15,000 *g* for 5 min, and the supernatant was filtered using a 0.45 μm polyvinylidene fluoride (PVDF) membrane filter (Millipore; Bengaluru, Karnataka, India). The filtrate was collected and diluted with 50 *v*/*v*% methanol. The dissolved amount of DTX was analyzed using HPLC. Solubility enhancing capacity per unit quantity (SEC) was calculated with the following equation [4]:

$$SEC\ \ (\%/mL) = 100 \times \frac{SOL_{DTX@PLC} - SOL_{DTX}}{Mass_{phospholipid}}$$

where $SOL_{DTX@PLC}$ and Sol_{DTX} represent the solubility of DTX@PLC and DTX material, respectively, $Mass_{phospholipid}$ represents the amount (mg) of added phospholipid.

1.4. Characterization of DTX@PLC

1.4.1. X-ray Diffraction (XRD)

The crystalline state of DTX (raw material), phospholipid, physical mixture, and DTX@PLC were measured using D/Max-2000 Ultima/PC (Rigaku, Tokyo, Japan) The scan range (2θ) was 5–60°, with scanning size of 0.02 °/s.

1.4.2. Fourier Transform Infrared Spectroscopy (FT-IR)

In order to clarify the chemical interaction between DTX and phospholipid, Fourier transform infrared spectroscopy (FT-IR) spectra of DTX (raw material), phospholipid, physical mixture, and

DTX@PLC were investigated with Thermo Scientific Nicolet 380 Spectrophotometer (Thermo Fisher Scientific, Waltham, MA, USA). The spectrum was collected in the wavelength range of 4000–500 cm^{-1}.

2. Supplementary Results and Discussion

2.1. Prepaation and Characterization of DTX@PLC

Figure S1 shows that the solubility of DTX increased to 1.4 mg/mL as the mass ratio of phospholipid increased. As the phospholipid formed a complex with DTX, the solubility of DTX was improved. On the other hand, SEC showed the highest value at a mass ratio of 1:3, which means that the efficiency of DTX@PLC was the best at the mass ratio. Therefore, a mass ratio of 1:3 was chosen for further studies.

2.2. Physicochemical Characterizations of DTX@PLC

Figure. S2A showed the X-ray diffraction (XRD) patterns of DTX (raw material), phospholipid, physical mixture, and DTX@PLC. The diffraction pattern of raw material showed sharp crystalline peaks at 8.8°, 11.1°, 14.0°, and 17.7°. In the case of phospholipid, the crystalline peaks at 5.8° and 7.6°. Most of the peaks were also detected in physical mixture. However, peaks indicating crystallinity were not detected in DTX@PLC, suggesting that the crystal form of DTX and phospholipid was transformed into an amorphous/solubilized form through complexation.

The chemical interaction between DTX and phospholipid was investigated using FT-IR. The FT-IR spectra of DTX (raw material), phospholipid, physical mixture, and DTX@PLC were depicted in Figure. S2B. DTX exhibited characteristic peaks at 1710 and 707 cm^{-1}. Phospholipid showed specific peaks at 2923, 2854, 1734, and 1062 cm^{-1}. In DTX@PLC, overlapped peaks of DTX and phospholipid were observed without spectral shift, indicating no interaction between DTX and phospholipid. The overall spectrum of DTX@PLC was similar to that of physical mixture. These results indicated that the functional group of DTX was invariant by complexation, suggesting that DTX could be complexed with phospholipids while maintaining the original characteristics of DTX.

3. Supplementary Tables

Table S1. Coefficient equations of responses according to the level of factors.

Responses	Coefficient Equations
Y_1	$76.16X_1 + 23.96X_2 + 83.63X_3$
Y_2	$256.70X_1 + 3.39X_2 + 90.84X_3 - 241.88X_1X_2 - 206.44X_1X_3 + 159.56X_2X_3 - 2982.19X_1^2X_2X_3 + 3284.48X_1X_2^2X_3 - 271.23X_1X_2X_3^2$
Y_3	$560.19X_1 + 52.23X_2 + 626.68X_3$
Y_4	$5900.79X_1 + 99.75X_2 + 10.36X_3 - 10526.98X_1X_2 - 10450.65X_1X_3 - 119.17 X_2X_3 + 10294.54X_1X_2X_3 - 5115.64X_1X_2(X_1 - X_2) - 5099.69X_1X_3(X_1 - X_3) - 65.38X_2X_3(X_2 - X_3)$

Table S2. Analysis of variance for model of solubility (Y_1).

Source	df	Mean Square	*F*-value	*p*-value
Model	2	3739.03	221.42	<0.0001
Linear Mixture	2	3739.03	221.42	<0.0001
Residual	14	16.89		
Lack of fit	9	22.90	3.77	0.0789
Pure error	5	6.07		
Total	16			

Note: df (Degree of freedom), a large *F*-value implies a large impact on the modeling profile; a *p*-value of less than 0.05 means that it affects modeling profile.

Table S3. Analysis of variance for model of precipitation (Y_2).

Source	df	Mean Square	*F*-value	*p*-value
Model	8	2640.85	77.21	<0.0001
Linear Mixture	2	7720.70	225.73	<0.0001
X_1X_2	1	39.31	1.15	0.3150
X_1X_3	1	28.63	0.8372	0.3869
X_2X_3	1	2137.21	62.49	<0.0001
$X_1^2X_2X_3$	1	91.35	2.67	0.1408
$X_1X_2^2X_3$	1	625.10	18.28	0.0027
$X_1X_2X_3^2$	1	4.26	0.1246	0.7332
Residual	8	34.20		
Lack of fit	3	47.86	1.84	0.2571
Pure error	5	26.01		
Total	16			

Note: df (Degree of freedom), a large *F*-value implies a large impact on the modeling profile; a *p*-value of less than 0.05 means that it affects modeling profile.

Table S4. Analysis of variance for model of droplet size (Y_3).

Source	df	Mean Square	*F*-value	*p*-value
Model	2	3.469×10^5	19.52	<0.0001
Linear mixture	2	3.469×10^5	19.52	<0.0001
Residual	14	17767.52		
Lack of fit	9	20977.98	1.75	0.2788
Pure error	5	11988.70		
Total	16			

Note: df (Degree of freedom), a large *F*-value implies a large impact on the modeling profile; a *p*-value of less than 0.05 means that it affects modeling profile.

Table S5. Analysis of variance for model of transmittance (Y_4).

Source	df	Mean Square	*F*-value	*p*-value
Model	9	1871.70	284.41	<0.0001
Linear mixture	2	7232.50	1098.99	<0.0001
X_1X_2	1	375.36	57.04	0.0001
X_1X_3	1	369.94	56.21	0.0001
X_2X_3	1	1249.71	18989	<0.0001
$X_1X_2X_3$	1	402.44	61.15	0.0001
$X_1X_2(X_1 - X_2)$	1	409.74	62.26	< 0.0001
$X_1X_3(X_1 - X_3)$	1	407.51	61.92	0.0001
$X_2X_3(X_2 - X_3)$	1	51.26	7.79	0.0269
Residual	7	6.58		
Lack of fit	2	2.77	0.3416	0.7260
Pure error	5	8.11		
Total	16			

Note: df (Degree of freedom), a large *F*-value implies a large impact on the modeling profile; a *p*-value of less than 0.05 means that it affects modeling profile.

Table S6. Correlation coefficient (R^2) values of docetaxel (DTX)-formulations in various dissolution models.

Media	Formulations	R^2				
		**Zero-order**	**First-order**	**Higuchi**	**Hixson-Crowell**	**Korsmeyer-Peppas**
pH 1.2	DTX	0.9547	0.9643	**0.9849**	0.9613	0.9039
	DTX@PLC	0.5168	0.5554	0.7609	0.5427	**0.9369**
	DTX-SME	0.6958	0.8790	0.9073	0.8246	**0.9548**
	DTX@PLC-SME	0.6335	0.8821	0.8699	0.8078	**0.9286**
pH 4.0	DTX	0.9008	0.9194	**0.9859**	0.9134	0.9390
	DTX@PLC	0.7943	0.9439	0.9343	0.8277	**0.9469**
	DTX-SME	0.6585	0.8338	0.8580	0.7736	**0.9609**
	DTX@PLC-SME	0.6838	0.9148	0.8756	0.8400	**0.9588**
pH 6.8	DTX	0.9338	0.9471	**0.9949**	0.9428	0.9504
	DTX@PLC	0.7725	0.8228	0.9277	0.8064	**0.9602**
	DTX-SME	0.5720	0.7612	0.8184	0.6986	**0.9148**
	DTX@PLC-SME	0.5539	0.7765	0.,8055	0.7027	**0.9107**
Distilled water	DTX	0.8027	0.8205	0.9578	0.8147	**0.9704**
	DTX@PLC	0.6838	0.9363	0.8775	0.9680	**0.9706**
	DTX-SME	0.5631	0.7460	0.7980	0.6830	**0.9262**
	DTX@PLC-SME	0.5566	0.7565	0.7978	0.6882	**0.9188**

Results

To investigate the mechanism of drug release, various dissolution models (zero-order, first-order, Higuchi, Hixson-Crowell, and Korsmeyer-Peppas models) were fitted to the release profiles of the formulations. Table S6 provides the R^2 values for all dissolution conditions tested, and the drug release mechanism was classified according to this value. As shown in the R^2 values, Korsmeyer-Peppas model fits well with the dissolution profiles of DTX@PLC, DTX-SME, and DTX@PLC-SME. In the case of DTX, most of the media, except for distilled water, followed Higuchi model.

Table S7. IC_{50} values of docetaxel (DTX), docetaxel-phospholipid complex (DTX@PLC), docetaxel-loaded self-microemulsifying drug delivery system (DTX-SME), and docetaxel-phospholipid complex loaded self-microemulsifying drug delivery system (DTX@PLC-SME), determined by MTT assay. Values are represented as mean ± SD (n = 6).

Samples	DTX	DTX@PLC	DTX-SME	DTX@PLC-SME
IC_{50} (μg/mL)	35.8 ± 9.8	39.1 ± 8.1	26.9 ± 5.2	26.4 ± 5.1

Table S8. Long-term stability of DTX@PLC-SME (n = 3).

Long-term Stability	0 Week (initial)	1 Week	2 Weeks	3 Weeks	4 Weeks
Droplet size (nm)	117.1 ± 6.7	125.4 ± 6.9	112.8 ± 7.2	124.1 ± 8.1	129.7 ± 8.9
Precipitation (%)	8.9 ± 2.6	8.7 ± 1.8	9.2 ± 2.1	9.1 ± 2.4	8.8 ± 1.4
Drug content (%)	99.6 ± 1.3	99.2 ± 0.6	98.9 ± 1.1	99.1 ± 0.9	99.5 ± 1.4

Table S9. Freeze-thaw cycle stability of DTX@PLC-SME (n = 3).

Freeze-Thaw	0 Cycle (initial)	1 Cycle	2 Cycle	3 Cycle
Droplet size (nm)	117.1 ± 6.7	114.7 ± 3.1	122.8 ± 2.6	118.5 ± 5.3
Precipitation (%)	8.9 ± 2.6	7.9 ± 3.2	9.1 ± 3.4	9.0 ± 2.2
Drug content (%)	99.6 ± 1.3	98.7 ± 3.7	99.4 ± 1.1	99.3 ± 2.6

Table S10. Heating-cooling cycle stability of DTX@PLC-SME (n = 3).

Heating-Cooling	0 Cycle (initial)	1 Cycle	2 Cycle	3 Cycle
Droplet size (nm)	117.1 ± 6.7	121.3 ± 5.7	124.6 ± 5.7	119.7 ± 4.8
Precipitation (%)	8.9 ± 2.6	8.4 ± 2.4	9.3 ± 3.4	9.1 ± 2.8

Drug content (%)	99.6 ± 1.3	99.1 ± 2.2	98.4 ± 1.6	98.9 ± 2.1

Methods

Long-term stability

The stability of DTX@PLC-SME was investigated for 4 weeks. DTX@PLC-SME was stored in glass vials capped at 25 °C for 4 weeks. The samples (100 mg) were added to 10 mL of distilled water, and then dispersed to form a homogeneous emulsion. At predetermined time-points, physical properties (particle size, precipitation, and drug content) were evaluated.

Freeze-thaw cycle stability

To test the freeze-thaw cycle stability, three freeze-thaw cycles between –20 and 25 °C were performed on DTX@PLC-SME, stored at each temperature for at least 24 h. The samples (100 mg) were added to 10 mL of distilled water and then dispersed to form a homogeneous emulsion. At predetermined time-points, physical properties (particle size, precipitation, and drug content) were evaluated.

Heating-cooling cycle stability

The cycle of storing for more than 24 h at 4 to 45 °C was studied three times. The samples (100 mg) were added to 10 mL of distilled water and then dispersed to form a homogeneous emulsion. At predetermined time-points, physical properties (particle size, precipitation, and drug content) were evaluated.

4. Supplementary Figures

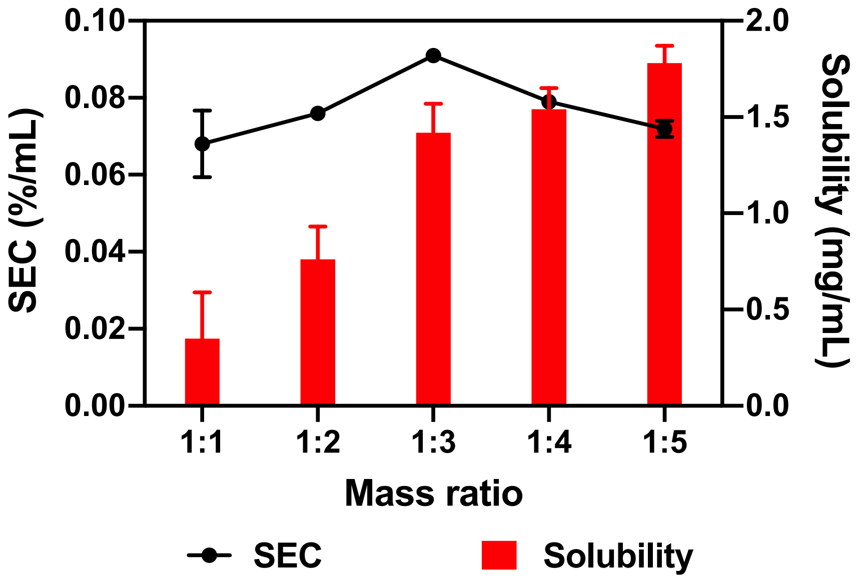

Figure S1. Solubility and solubility enhancing capacity (SEC) versus mass ratio (docetaxel (DTX):phospholipid). Values are presented as mean ± SD (n = 3).

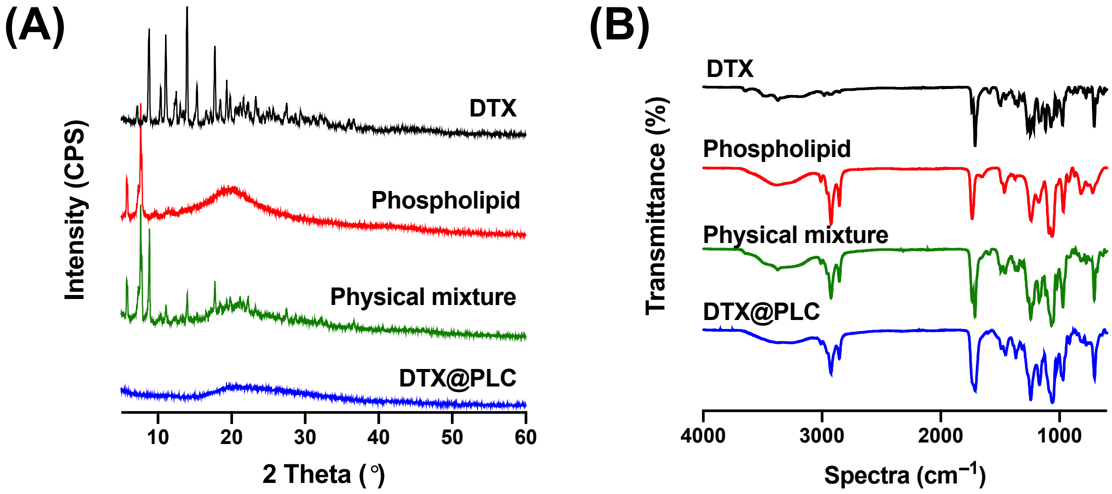

Figure S2. (**A**) X-ray diffraction (XRD) patterns and (**B**) Fourier transform infrared spectroscopy (FT-IR) spectra of DTX, phospholipid, physical mixture, and docetaxel-phospholipid complex (DTX@PLC).

.

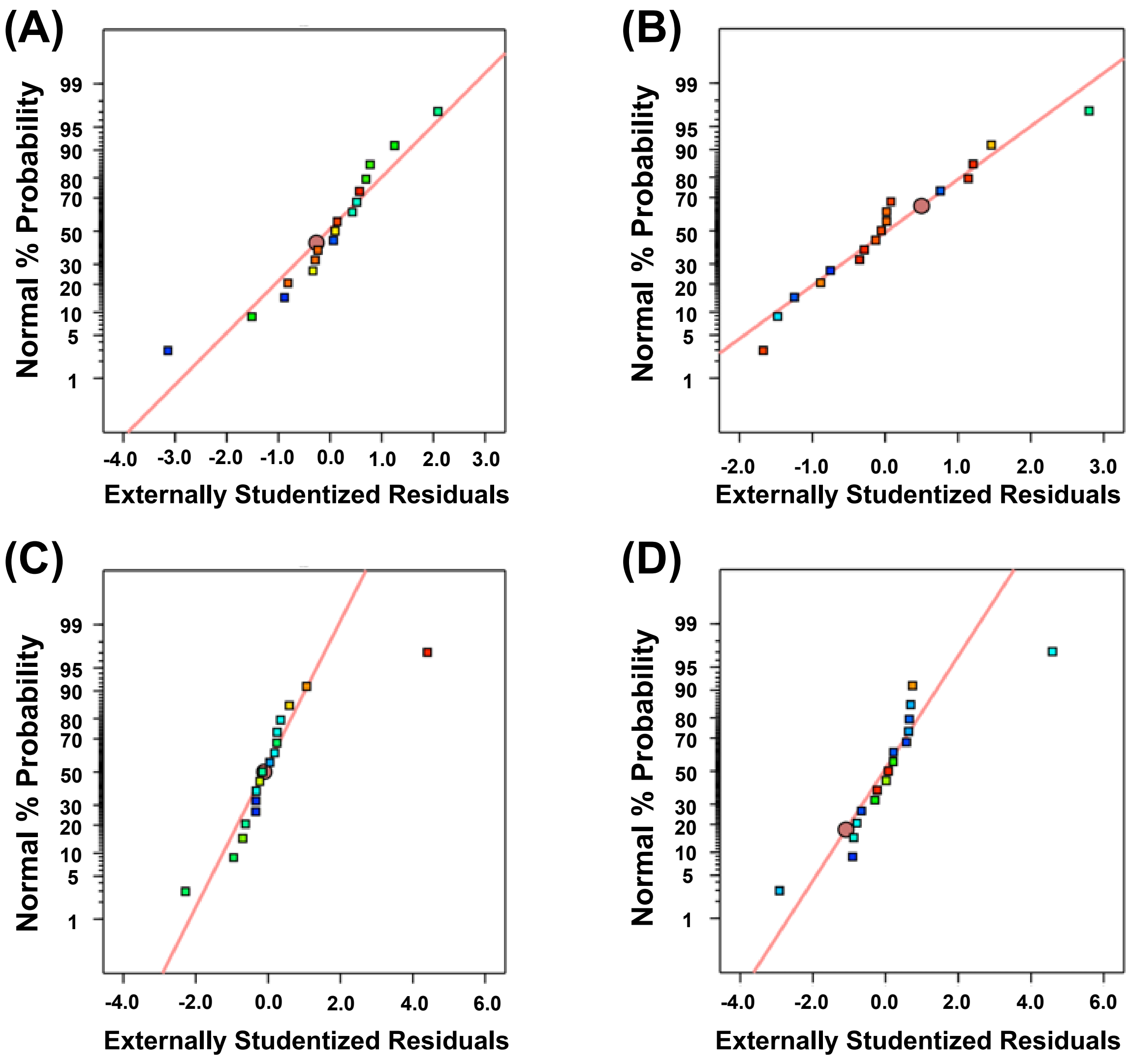

Figure S3. Residual plots. (**A**) Y_1: solubility; (**B**) Y_2: precipitation; (**C**) Y_3: droplet size; (**D**) Y_4: transmittance.
